# Synthesis, Structure, and Properties of Polyhydroxybutyrate Derived from *Azotobacter Vinelandii* N‐15

**DOI:** 10.1002/open.202500150

**Published:** 2025-05-06

**Authors:** Yang Liu, Dongwei Li, Xianghai Ran, Wei Nie, Ihor Semeniuk, Nataliia Koretska

**Affiliations:** ^1^ CAS Key Laboratory of high‐promance Synthetic Rubber and its Composite Materials Changchun institute of applied chemistry Chinese Academy of Sciences 5625 Renmin. Ave Changchun Jilin 130000 China; ^2^ L. M. Lytvynenko of the National Academy of Sciences of Ukraine Department of Physical Chemistry of Fossil Fuels 3a, Naukova Str. Lviv 79060 Ukraine

**Keywords:** analytical methods, molasses, polyhydroxyalkanoates, polyhydroxybutyrate, polymers

## Abstract

Biodegradable polymers are of great interest in addressing the current pollution problem caused by synthetic petroleum‐based polymers. It is well known that various microorganisms synthesize and store high‐molecular‐weight polyhydroxyalkanoates in their cytoplasm as water‐insoluble inclusions. In this study, the bacterium *Azotobacter vinelandii* N‐15 strain is used for bioplastic production. The optimal polyhydroxybutyrate (PHB) yield (62% of biomass, 23.6 g L^−1^ dry cells) is achieved by cultivating the bacteria in Burke's medium with molasses as a carbon source (5 wt.%) at 30 °C, 220 rpm, for 50 h. The resulting polymer was characterized using thin‐layer chromatography, UV‐Vis, fourier transform infrared, nuclear magnetic resonance spectroscopy, gas chromatography, and X‐ray diffraction. The results confirmed that the polymer is PHB with a purity of 98.9%, a molecular weight of 1.2 MDa, a crystallinity of 73%, a melting point of 179 °C, a decomposition temperature of 275 °C, a density of 1.22 g cm^−^^3^, a melt flow index of 10 g 10 min^−1^, a Shore hardness of 82, a tensile strength of 30 MPa, and a relative elongation at break of 4.5%. Thus, a bioplastic with properties suitable for practical applications is successfully obtained using molasses—a byproduct of sugar production.

## Graphical abstract

The bacterium *Azotobacter vinelandii* N15 synthesizes polyhydroxybutyrate (PHB) from sugar production waste. The study reveals the molecular composition, crystallinity, and thermal behavior of the synthesized biopolymer. Mechanical tests demonstrated high tensile strength and relatively low elongation at break. These properties highlight PHB's potential as a sustainable material for industrial applications, capable of replacing petroleum‐based polymers.

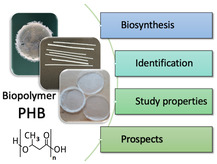

## 1 Introduction

Plastics are an integral part of modern everyday life. They are widely used due to their low cost, durability, and favorable mechanical and thermal properties.^[^ ^1^, ^2^ ^]^ Derived from petrochemical raw materials, conventional plastics are highly durable but non‐biodegradable. ≈140 million tons of plastic are produced globally each year, with a significant portion accumulating in the environment, posing serious risks to ecosystems and human health.^[^ ^1^, ^3^ ^]^

The challenges associated with plastic waste disposal, along with the depletion of global oil resources, have driven the development of technologies for producing biodegradable polymers from natural sources.^[^ ^4^, ^5^ ^]^

Biodegradable polymer‐based plastics offer several advantages in the transition to a circular economy. Their use supports more sustainable life cycles for commercial plastics. Compared to fossil‐based plastics, bioplastics can have a smaller carbon footprint and exhibit beneficial material properties. Additionally, bioplastics may be compatible with existing recycling processes or be biodegradable. However, these benefits often come with trade‐offs, such as higher production costs.^[^ ^6^ ^]^ Nevertheless, ongoing technological advancements aim to minimize these drawbacks.

Bioplastics are derived from biopolymers such as polyhydroxyalkanoates (PHAs), which are produced by various bacteria and can undergo both aerobic and anaerobic degradation.^[^ ^7^, ^8^ ^]^

PHAs can be synthesized from renewable carbon sources, particularly industrial waste. They accumulate in bacterial cells, especially when grown in unbalanced media, and consist of 600–35 000 monomeric units of hydroxyalkanoic acid.^[^ ^9^ ^]^ Each monomeric unit contains a side‐chain group (R), which is typically a saturated alkyl group.^[^ ^7^ ^]^ Depending on their chemical composition, PHAs exhibit varying properties, yet all are insoluble in water and resistant to hydrolysis and UV radiation.^[^ ^10^ ^]^

PHAs possess favorable physical and mechanical properties, making them suitable replacements for conventional polymers in many applications. At the same time, they are biodegradable and biocompatible.^[^ ^7^, ^9^ ^]^ This unique combination of characteristics opens up opportunities for innovative applications.^[^ ^8^ ^]^

PHAs have significant practical potential in medicine, where they are used for implants, tendon and cartilage elements, sutures for wound closure, and drug delivery systems.^[^ ^11^ ^]^ In agriculture, PHAs are employed for seed and fertilizer encapsulation, greenhouse plant protection films,^[^ ^12^ ^]^ and as biodegradable packaging materials.^[^ ^13^, ^14^ ^]^

**Figure 1:**
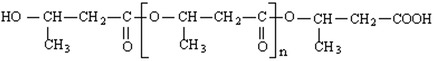
Structural formula of polyhydroxybutyrate.^[^ ^16^ ^]^

The most extensively studied PHA is polyhydroxybutyrate (PHB), first discovered by Lemoine in 1923 as a component of the bacterium *Bacillus megaterium*.^[^ ^15^ ^]^ PHB is a homopolymer of D‐3‐hydroxybutyrate (**Figure** **1**). It is completely isotactic and has no branched chains.^[^ ^16^ ^]^

Among its valuable characteristics are a high degree of crystallinity, relative resistance to hydrolytic degradation, piezoelectricity, optical activity, biodegradability, and biocompatibility. Synthesized by bacteria from renewable raw materials, PHB is an eco‐friendly, highly hydrophobic, partially crystalline thermoplastic polyester.^[^ ^7^, ^8^ ^]^ Since PHB synthesis is entirely microbial, it does not contain catalyst residues, unlike other synthetic polymers. PHB holds broad prospects for application in various industries, including agriculture, electronics, medicine, the food industry, packaging, and more.^[^ ^11^, ^12^, ^13^ ^]^

Despite the large number of scientific studies, the problem of microbial PHB synthesis remains relevant, as evidenced by the increasing number of publications each year.^[^ ^17^ ^]^ Bacteria of the genus *Azotobacter* are aerobic soil microorganisms that can fix atmospheric nitrogen and are among the promising producers of PHB.^[^ ^18^ ^]^ These bacteria can assimilate various carbon sources. However, sugars (such as sucrose, glucose, and fructose) are commonly used for PHB biosynthesis,^[^ ^18^ ^]^ and current research is focusing on utilizing industrial waste (e.g., technical glycerin,^[^ ^19^ ^]^ sugar cane juice,^[^ ^20^ ^]^ fruit residues,^[^ ^21^ ^]^ and molasses).^[^ ^22^ ^]^ Information on PHB synthesis from industrial waste is available but often limited and not comprehensive.

Molasses, a byproduct of sugar production, is a thick, viscous liquid with a brown or dark brown color. It has a characteristic odor, a sweet taste, and a bitter aftertaste. Molasses contains sugar impurities (sucrose, glucose, and fructose) and non‐sugar impurities, including nitrogenous compounds, aliphatic free and bound acids, trace elements, vitamins, and growth regulators.^[^ ^23^ ^]^ Mineral salts in molasses provide microorganisms with essential ions, such as potassium, ferrous iron, calcium, magnesium, phosphate, and sulfate, as well as trace elements like zinc, cobalt, manganese, copper, and molybdenum.^[^ ^23^ ^]^ Thus, molasses is an attractive raw material for bioplastic production, owing to its suitable composition and low cost.

At the same time, studying the physical and mechanical properties of PHB is important, as it broadens its potential for practical applications.

Therefore, the aim of this work is to investigate the dynamics of polyhydroxybutyrate biosynthesis by the *A. vinelandii* N‐15 strain, identify the polymer, and evaluate its properties to determine its practical potential.

## 2 Experimental Section

### 2.1 Materials

*The Azotobacter vinelandii* strain N‐15 (from the collection of Daughter enterprise “ENZIM”, Vinnytsia, Ukraine) was used for the synthesis of polyhydroxybutyrate. Beet molasses with the following composition (wt%): sucrose ‐ 32, glucose ‐ 14, fructose ‐ 16, water ‐ 22, and non‐sugar impurities – 16, was obtained from PJSC Enzyme (Lviv, Ukraine). Salts, nutrient media, and solvents were purchased from Sigma‐Aldrich.

### 2.2 Methods

#### 2.2.1 Bacterial Cultivation

The cultivation of *A. vinelandii* N‐15 bacteria was carried out in Erlenmeyer flasks (750 mL) with a filling factor of 0.14 on a rotary shaker (220 rpm) at a temperature of 30 ± 0.5 °C, pH 6.9–7.2, for 70 h. For the growth of bacteria, a modified Burke's nutrient medium^[^ ^24^ ^]^ was used. The composition of modified Burke's nutrient medium was as follows (g/L): K_2_HPO_4_ × 3H_2_O – 0.8; KH_2_PO_4_ – 0.2; NaCl – 0.5; CaSO_4_ – 0.13; MgSO_4_ × 7H_2_O – 0.2; molasses – 40, 50, or 60.

The inoculum of *A. vinelandii* N‐15 strain was grown on enriched Ashby's nutrient medium^[^ ^25^ ^]^ supplemented with mannitol and 1 mL L^−1^ of the trace element solution. The composition of nutrient medium, g L^−1^: K_2_NPO_4_ – 0.2; MgSO_4_ – 0.2; NaCl – 0.2; K_2_SO_4_ – 0.1, Na_2_MoO_4_ × 2H_2_O – 0.006; CaCO_3_ – 5.0; mannitol – 30. The composition of solution of trace elements, g L^−1^: FeSO_4_ × 7H_2_O–0.05; ZnSO_4_ × 7H_2_O – 0.2; H_3_BO_3_ – 0.6; MnCl_2_ × 4H_2_O – 0.06; CoCl_2_ × 6H_2_O – 0.4; CuSO_4_ × 4H_2_O – 0.02; NaMoO_4_ × 2H_2_O – 0.01. A 24‐h culture from the exponential growth phase (2 × 10^8^ CFU/cm^3^, 10 vol%) was used as an inoculum.

#### 2.2.2 Determination of Biomass and PHB Content in Cells

Every 10 h, the culture fluid was collected and centrifuged at 8000 rpm for 15 min. The resulting precipitate was washed twice with NaCl solution (0.9 wt%) and dried at 60 °C to a constant weight. Biomass was determined using an analytical weighing scale (KERN ADB 100‐4, 120 G/0.0001 G).

For determination of PHB content, dry cells were lysed with ethanol (20 min, 30 °C) and ultrasound using an ultrasonic bath (GT SONIC VGT‐6250, China). After centrifugation (15 min, 6000 rpm), the precipitate was extracted for five cycles in a Soxhlet apparatus with chloroform. The resulting solution was concentrated. The polymer was precipitated with ethyl alcohol and then centrifuged (15 min at 6000 rpm). For purification, the polymer was dissolved in chloroform and precipitated again with ethyl alcohol. The resulting PHB was purified from solvent residues by vacuum evaporation at 60 °C to constant weight.^[^ ^14^ ^]^

#### 2.2.3 Thin‐Layer Chromatography (TLC)

TLC was performed on a Merck TLC Silicagel plate (Germany) using an ethyl acetate‐benzene (1:1) solvent system. Visualization was carried out in an iodine chamber for 5–10 min. The appearance of yellow‐brown spots with an Rf value of 0.8 indicated the presence of PHA.^[^ ^26^, ^27^ ^]^

#### 2.2.4 Infrared Spectroscopy (IR)

To analyze the presence of functional groups in the polymer structure, a Bruker VERTEX 70 Fourier transform infrared spectrometer (Germany) was used. The wave number range was 4000–400 cm^−1^, with a resolution of 4 cm^−1^ and 128 scans. A diamond reflective crystal was used.^[^ ^28^ ^]^ Literature data^[^ ^29^ ^]^ were used for spectrum interpretation.

#### 2.2.5 Ultraviolet‐Visible Spectroscopy (UV‐Vis)

UV‐Vis analysis was conducted according to the method described by Sato et al.^[^ ^30^ ^]^ The purified and dried polymer (20 mg) was transferred to a test tube, and 10 mL of concentrated H_2_SO_4_ was added. The solution was heated in a water bath for 10 min. After cooling and mixing, the absorption spectrum of the resulting solution was recorded. Concentrated H_2_SO_4_ was used as a solvent for comparison. The absorption spectrum was analyzed in the range of 200–800 nm using a quartz cuvette (1 mm) and a UV mini‐1240 spectrophotometer (Shimadzu, Japan).

#### 2.2.6 ^1^H NMR Spectroscopy

The composition of hydroxyalkanoate units in the polymer was determined by analyzing the nuclear magnetic resonance (NMR) spectra. The proton (^1^H) NMR spectra of the obtained biopolymer, dissolved in deuterated chloroform, were recorded using a Bruker AVANCE III spectrometer operating at a frequency of 500 MHz (AV500). Tetramethylsilane was used as an internal standard. The test temperature was 25 °C. Samples were prepared by dissolving chloroform‐cast film segments in deuterated chloroform (2% w v^−1^). NMR spectral data were processed using MestReNova Version software.

#### 2.2.7 X‐ray Diffraction (XRD)

**Figure 2:**
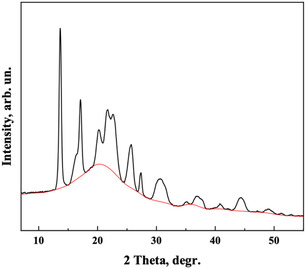
Sample diffractogram with a baseline separating crystalline peaks from the amorphous region.^[^ ^31^ ^]^

XRD analysis was performed using an AERIS Research X‐ray Diffractometer (Malvern PANalytical). Crushed and milled polymer samples were analyzed. X‐ray diffractograms were recorded at 25 °C in the range of 10°–40° (2*θ*) with a step size of 0.022° and a scan time of 24 s. The degree of crystallinity of the samples was determined using OriginPro software (OriginLab Corporation, Northampton) based on the obtained data. The degree of crystallinity was calculated as the ratio of the total integrated area under the crystalline peaks to the total integrated area under the X‐ray scattering curve. For this calculation, a baseline was drawn from the start to the end of the curve and through the bases of each peak (**Figure** **2**).

This baseline divides the curve into crystalline and amorphous regions. The degree of crystallinity (DC) was calculated using the following formula^[^ ^31^ ^]^

(1) $$D_{C}=\frac{\Sigma A_{c}}{\Sigma \left(A_{c}+A_{a}\right)}$$

where *A*_c_ is the integral area under crystalline peaks, and *A*_a_ is the integral area under the amorphous halo.

#### 2.2.8 Gas Chromatography (GC)

A mixture containing 2 mg of the polymer under study, 2 mL of chloroform, 1.8 mL of methanol, and 0.3 mL of sulfuric acid was heated at 100 °C for 140 min. This process led to the polymer breakdown via methanolysis, forming β‐hydroxycarboxylic acid methyl esters. After cooling to room temperature, the mixture was washed with 2 mL of water and vigorously shaken for 1 min with a 10–12% NaHCO_3_ solution. After phase separation, the organic phase (bottom layer) was removed, dried over anhydrous Na_2_SO_4_, and filtered through a 0.45 μm nylon syringe filter. The product was then transferred to a small glass tube with a screw cap and stored in a freezer at −18 °C until further analysis.^[^ ^32^ ^]^

Before analysis, methyl benzoate (5 mg L^−1^) was added as an internal standard. The analysis was performed by injecting 1 μL of the prepared solution into a Thermo Trace 1300 Series Gas Chromatograph (Thermo Fisher Scientific S.p.A., Milan, Italy) equipped with an FID detector and a WM‐FFAR capillary column (30 m × 0.32 mm × 0.25 mm). The maximum operating temperature was 240 °C.

Conditions for the chromatographic separation of polyhydroxybutyrate: detector FID, evaporator temperature 230 °C, initial column temperature (60 °C) was maintained for 5 min and then increased at a rate of 10 °C min^−1^ to 220 °C, detector temperature 275 °C. Standardization was performed under the same conditions using analytical PHBs (BIOMER).

The amount of methyl ester obtained via gas chromatography reflected the PHB content in the sample.

#### 2.2.9 Gel Permeation Chromatography (GPC)

The weight‐average molecular weight (Mw), number‐average molecular weight (Mn), and polydispersity index (Mw/Mn) of the purified polymer were determined using gel permeation chromatography.

The analysis was conducted using a PL‐GPC120 gel permeation chromatography system connected to a refractive index detector (Polymer Labs PL‐BV 400) and equipped with PLgel MIXED‐B LS columns (300 × 7.5 mm × 2). A polymer solution in HPLC‐grade chloroform (1 mg mL^−1^) was filtered through a 0.45 μm PTFE membrane. The eluent was chloroform, with a flow rate of 0.8 mL min^−1^ at 30 °C. Universal calibration was performed using polystyrene standards (Agilent, USA).

The calibration curve was correlated with PHB using the Mark–Houwink–Sakurada equation
(2)

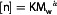

where [*η*] is the intrinsic viscosity, and K and α are the Mark–Houwink constants for each polymer/solvent/temperature system. For PHB in chloroform, K = 0.0118 mL g^−1^, *ά* = 0.78; for polystyrene in chloroform, K = 0.0049 mL g^−1^, *ά* = 0.78.^[^ ^33^ ^]^ The injection volume was 150 μL.

#### 2.2.10 Thermogravimetric Analysis (TGA)

Thermal analysis was performed using a Paulik–Paulik–Erdey Q‐1500D derivatograph in the temperature range of 20–800 °C under free air access. The heating rate was 5 °C min^−1^, with an average polymer weight of 100 mg. Aluminum oxide was used as the reference substance.^[^ ^34^ ^]^

#### 2.2.11 Physico‐Mechanical Properties

**The melt flow index** was determined according to ASTM D1238 using an IRT‐A plastometer (190 °C, under a 2.16 kg load). Cylindrical PHB samples were prepared using a vertical injection molding machine MLV‐32 (175 °C, 0.5 MPa, 10 min). The density of the samples was determined according to ASTM D792.

**Shore D hardness** was measured in accordance with ASTM D2240 using a Shore D Durometer digital hardness tester under a 5 kg load.

**Tensile strength and elongation at break** were determined for the films. PHB films were prepared by dissolving the polymer in chloroform (3% w v^−1^) at 70 °C under stirring for 1 h. The resulting solutions were applied to a horizontally mounted silica glass plate using an AU1‐65 applicator, followed by solvent evaporation at room temperature for 48 h. The dried films (thickness: 50 ± 5 μm) were further vacuum‐dried at 60 °C for 2 h in a VS‐0.035 vacuum oven to remove residual solvent. For testing, a Kimura Machinery RT‐601U universal testing machine was used (tensile speed: 50 mm min^−1^). Mechanical properties were determined according to ASTM D638, including tensile strength and elongation at break.

#### 2.2.12 Statistical Analysis

Each sample was measured five times. Results are presented as arithmetic means with standard deviations. Statistical data processing was performed using standard Microsoft Excel methods.

## 3 Results and Discussion

### 3.1 Cell Growth and PHB Accumulation

The bacterium *Azotobacter vinelandii* N‐15 grows efficiently on molasses, a byproduct of the sugar industry. The use of molasses enables PHB biosynthesis without the need for additional trace elements,^[^ ^23^ ^]^ resulting in high PHB yields and demonstrating the economic feasibility and industrial potential of this process.

**Figure 3:**
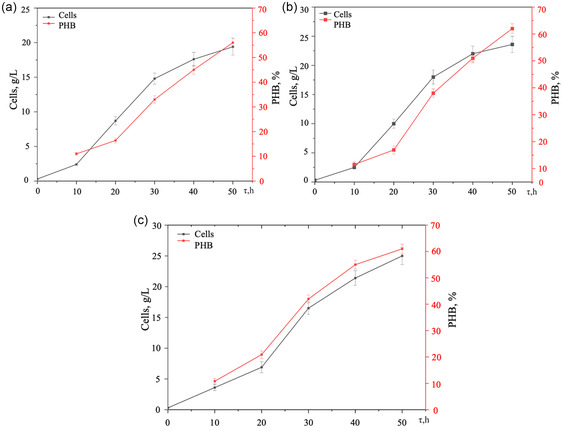
Parameters of cell accumulation and PHB content in biomass over 50 h at different molasses concentrations: a) 4%; b) 5%; c) 6%. Cells ‐ dry biomass weight (g L^−1^); PHB ‐ intracellular polymer (% w w^−1^ of dry cells).

The parameters of cellular biomass accumulation and PHB production are shown in **Figure** **3**.

After 50 h, the biomass concentration ranged from 19.4 to 25 g L^−1^, with PHB content between 56% and 62%. When using 5% and 6% molasses, the PHB yield was slightly different (14.6 and 15.3 g L^−1^, respectively), but this difference was within the margin of error. Thus, a molasses concentration of 5% for 50 h is optimal for PHB synthesis by this strain (yielding 14.6 g L^−1^, which constitutes 60% of the biomass). Beyond this time, significant polysaccharide synthesis^[^ ^35^ ^]^ makes cell and PHB isolation more difficult. Additionally, in the stationary growth phase, PHB accumulation slows, and its degradation begins over time.^[^ ^18^ ^]^ Therefore, 50 h is sufficient for strain cultivation.

The PHB yields obtained (56–62% w w^−1^) exceed most values reported in the literature (5–47% w w^−1^) for bacteria cultivated on sugars, glycerol, molasses, and sugarcane.^[^ ^14^, ^36^, ^37^ ^]^ However, high‐yield strains in combination with efficient techniques can achieve up to 78% PHB (e.g., fed‐batch fermentation with sugarcane juice).^[^ ^20^ ^]^ These findings indicate that *A. vinelandii* strain N‐15 is promising for PHB synthesis using molasses.

### 3.2 Thin‐Layer Chromatography

**Figure 4:**
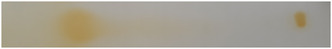
TLC of polyhydroxybutyrate from *A. vinelandii* N‐15 strain.

The identification of PHB via thin‐layer chromatography is shown in **Figure** **4**. The presence of a yellow‐orange spot with an R_f_ value of 0.8 confirms polyhydroxyalkanoate content, including PHB, consistent with literature data.^[^ ^26^, ^27^, ^38^ ^]^

### 3.3 UV‐Vis Spectroscopy

**Figure 5:**
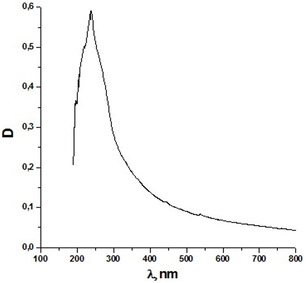
UV‐Vis spectrum of polyhydroxybutyrate from *A. vinelandii* N‐15 strain.

UV‐Visible spectroscopy revealed an absorbance maximum at 235 nm, indicating the presence of PHB **Figure** **5**.^[^ ^27^, ^30^ ^]^

### 3.4 Infrared Spectroscopy

**Figure 6:**
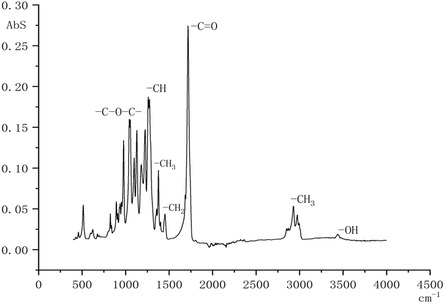
IR spectrum of polyhydroxybutyrate from *A. vinelandii* N‐15 strain.

Fourier transform infrared spectroscopy (FTIR) analysis (**Figure** **6**) identified a characteristic peak at 3440 cm^−1^ corresponding to the hydroxyl (‐OH) group, though present in small amounts. The absorption bands at 2928–2984 cm^−1^ indicate alkyl (‐CH_5_) groups, while the strongest band at 1726 cm^−1^ corresponds to C=O stretching.

The peak at 1280 cm^−1^ corresponds to a C—H group. The absorption bands in the 1185–1228 cm^−1^ range are characteristic of ester functional groups (C—*O*—C). Other absorption bands were also detected at 1363 and 1458 cm^−1^ for ‐CH_3_ and ‐CH_2_ groups, respectively.

The obtained results correspond to the functional groups present in the structure of PHB and agree with the result of Ramezani et al.^[^ ^27^ ^]^

### 3.5 ^1^ H NMR

**Figure 7:**
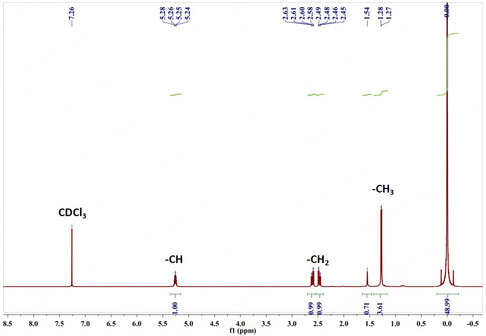
^1^H NMR spectrum of polyhydroxybutyrate from *A. vinelandii* N‐15 strain.

NMR spectroscopy is widely used for PHA characterization.^[^ ^16^ ^]^ The 1 H NMR spectrum (**Figure** **7**) displays three characteristic PHB signals: a doublet at *δ* = 1.27–1.28 ppm (methyl –CH_3_ coupled to one proton), a doublet of quadruplet at *δ* = 2.45–2.63 ppm (methylene –CH_2_ adjacent to an asymmetric carbon bearing a single proton), and a multiplet at δ = 5.24–5.28 ppm (methyne ‐CH). Additional signals at *δ* = 1.55 ppm (water) and *δ* = 7.25 ppm (chloroform) are also present. The results obtained confirm the identity of PHB, which is consistent with literature data.^[^ ^39^ ^]^

### 3.6 X‐ray Diffraction

The crystallinity of the obtained PHB was investigated by X‐ray diffraction analysis. Crystallinity affects the optical, mechanical, thermal, and chemical properties of polymers. For example, highly crystalline polymers are rigid, have high melting points, and are less susceptible to solvent penetration, while amorphous polymers are softer and more ductile, and melt more gradually when heated, and are more susceptible to solvent penetration. Therefore, the crystallinity of a polymer is an important characteristic for its practical use.

**Figure 8:**
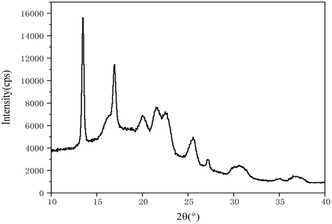
X‐ray diffractogram of polyhydroxybutyrate from *A. vinelandii* N‐15 strain.

The X‐ray diffractogram of PHB from *A. vinelandii* N‐15 strain revealed eight significant peaks at 2*θ* values of 13.45°, 16.92°, 20.18°, 21.53°, 22.53°, 25.49°, 27.25°, and 30.45° (**Figure** **8**). The calculated degree of crystallinity was 73%, exceeding reported PHB crystallinity values of 45–70%,^[^ ^40^ ^]^ similar to commercial PHBs (60–70%).^[^ ^41^ ^]^ The high crystallinity makes this biopolymer promising for industrial applications.

### 3.7 Gas Chromatography

Gas chromatography with flame ionization detection is widely used for PHB identification and quantification.

Comparison of the obtained results with literature data is valid only under similar gas chromatography conditions: the same type and size of the capillary columns, type of detectors, carrier gases, their flow rates, and temperature conditions.^[^ ^32^, ^42^ ^]^ The use of a standard PHB makes it possible to qualitatively determine the purity of the studied polymer without the need for further comparison with literature sources.

**Figure 9:**
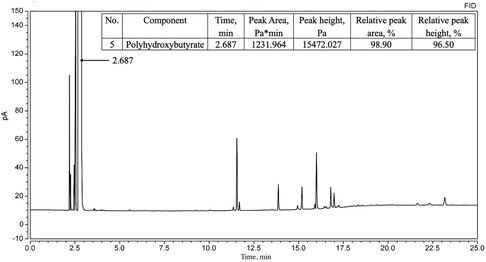
Chromatogram of methyl ester of β‐hydroxybutyric acid from the studied sample of *A. vinelandii* N‐15 strain.

**Figure 10:**
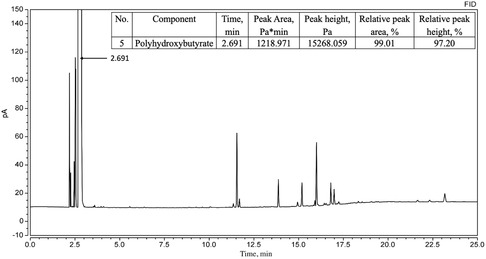
Chromatogram of methyl ester of β‐hydroxybutyric acid of standard PHB (BIOMER).

The chromatogram of PHB from *A. vinelandii* N‐15 strain (**Figure** **9**) displayed a peak at 2.687 min with 98.90% purity, closely matching the standard PHB (BIOMER) at 2.691 min (99.01% purity, **Figure** **10**). This confirms the high purity of the obtained PHB, comparable to commercial PHBs (89–99%).^[^ ^41^ ^]^

### 3.8 Gel Permeation Chromatography

**Gel permeation chromatography** was used to determine the molecular weight and polydispersity index of the obtained PHB. The weight‐average molecular weight (Mw) was 1.2 MDa, the number‐average molecular weight (Mn) was 0.466 MDa, and the polydispersity index (Mw/Mn) was 2.6. According to the literature, PHB molecular weights range from 0.177 to 6.6 MDa (Mw), with polydispersity indices between 1.2 and 2.5.^[^ ^43^, ^44^ ^]^ However, mutant strains can produce PHB with molecular weights (Mw) of up to 8 MDa.^[^ ^45^ ^]^ At the same time, commercially available PHB typically has an Mw of around 0.3 MDa, as the cost of production increases significantly with higher molecular weights.

### 3.9 Thermogravimetric Analysis

**Figure 11:**
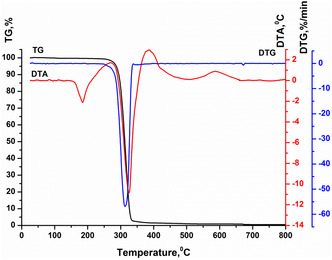
Thermogram of polyhydroxybutyrate from *A. vinelandii* N‐15 strain. TG ‐ thermogravimetric curve; DTA ‐ differential thermal analysis curve; DTG ‐ differential thermogravimetric curve.

The thermogravimetric curve (**Figure** **11**) revealed a melting point of 179 °C and thermal degradation starting above 275 °C. These properties indicate high thermal stability, making PHB suitable for practical applications.

The thermal properties of PHB produced by *A. vinelandii* N‐15 correlate with data reported in the literature.^[^ ^40^ ^]^

### 3.10 Physico‐Mechanical Properties

The physical and mechanical parameters of PHB produced by *A. vinelandii* N‐15 are presented in **Table** **1**. These properties were compared with those of conventional packaging plastics.

**Table 1:** Comparison of PHB properties produced by *a. vinelandii* strain N‐15 with some traditional plastics.

Physical and mechanical properties	Polymers						
	PHB of *A. vinelandii* N‐15	PP	LDPE	HDPE	PET	PS	References
Density, *ρ*, g cm^−3^	1.22 ± 0.05	0.91	0.92	0.95	1.35	1.05	[51, 52, 53]
Melting point, ^o^C	179	176	115	135	260	240	[51, 52]
Melt flow index, (MFI, g 10 min^−1^)	10.0 ± 1.0	3	0.3	0.5	32	6,5	[51, 52]
Shore hardness, HS (D), hardness units	82.0 ± 2.0	83	43	64	81	85	[51, 54]
Tensile strength, *σ*, MPa	30.0 ± 2.5	34.5	26	30.6	65	35	[51, 53]
Relative elongation at break, *ε*, %	4.5 ± 1.0	400	530	300	100	3.2	[51, 54]
Degree of crystallinity, %	73	50	40	85	35	68	[51, 54, 55]

PHB – polyhydroxybutyrate PP – polypropylene; LDPE – low‐density polyethylene; HDPE – high‐density polyethylene; PET – polyethylene terephthalate; PS – polystyrene.

Comparison of the properties of the obtained PHB with other polymers showed that PHB is most similar in its characteristics to polypropylene and polystyrene. Thus, one of the potential applications of PHB is to replace these synthetic polymers in various products. Improving the quality of PHB‐based materials can be achieved by creating composites with various compatible materials and other polymers. Thus, the relatively low elongation at break may limit its widespread use of PHB. This issue can be addressed by adding plasticizers of both synthetic and biogenic origin, such as microbial polymers like PHBV,^[^ ^46^ ^]^ 4‐PHB,^[^ ^47^ ^]^ polyhydroxyoctanoate,^[^ ^48^ ^]^ and polyhydroxydecanoate.^[^ ^49^ ^]^ There are also synthetic plasticizers on the market that are non‐toxic and biocompatible, such as HexamollDINCH‐ccycled.^[^ ^50^ ^]^

**Therefore, the potential applications of PHB** are not limited to packaging materials. Due to its biocompatibility, it holds significant promise for use in medicine, for example, in the production of implants, surgical sutures, and other biomedical devices.

## 4 Conclusions

The dynamics of PHB synthesis showed that *A. vinelandii* strain N‐15 grows best in Burke's nutrient medium supplemented with 5% molasses for 50 h, yielding 14.6 g L^−1^ of PHB, which constitutes 62% of the biomass.

The isolated polyhydroxybutyrate was identified using TLC, UV‐Vis spectroscopy, FTIR, NMR, and gas chromatography. The purity of the obtained PHB was 98.9%, and its degree of crystallinity was 73%. The molecular weight of the polymer (Mw = 1.2 MDa, Mn = 0.466 MDa) and its polydispersity index (Mw/Mn = 2.6) were also determined.

The heat resistance (275 °C) and thermal stability (179 °C) of the obtained PHB make it suitable for practical use in the production of household items such as packaging materials and containers.

Mechanical testing showed that the obtained PHB exhibits high tensile strength and relatively low elongation at break. Comparison with conventional polymers indicated that PHB is most similar in properties to polypropylene and polystyrene.

Moreover, the polymer has potential applications in medicine as a material for the manufacture of implants, surgical sutures, and other biomedical devices.

Thus, the use of molasses, a by‐product of sugar production, and *A. vinelandii* strain N‐15 enables the production of high‐purity PHB with favorable physical and mechanical properties. The results of this work can be used to develop innovative strategies for bacterial PHB production.

## Conflict of Interest

The authors declare no conflict of interest.

## Data Availability

The data that support the findings of this study are available from the corresponding author upon reasonable request.
